# Major Factors Affecting Incidence of Childhood Thyroid Cancer in Belarus after the Chernobyl Accident: Do Nitrates in Drinking Water Play a Role?

**DOI:** 10.1371/journal.pone.0137226

**Published:** 2015-09-23

**Authors:** Valentina M. Drozd, Vladimir A. Saenko, Alina V. Brenner, Vladimir Drozdovitch, Vasilii I. Pashkevich, Anatoliy V. Kudelsky, Yuri E. Demidchik, Igor Branovan, Nikolay Shiglik, Tatiana I. Rogounovitch, Shunichi Yamashita, Johannes Biko, Christoph Reiners

**Affiliations:** 1 The International fund “Help for patients with radiation-induced thyroid cancer “Arnica”, Minsk, Belarus; 2 Department of Endocrinology, Belarusian Medical Academy for Postgraduate Education, Minsk, Belarus; 3 Department of Radiation Molecular Epidemiology, Atomic Bomb Disease Institute, Nagasaki University, Sakamoto, Nagasaki, Japan; 4 Division of Cancer Epidemiology and Genetics, National Cancer Institute, National Institutes of Health, United States Department of Health and Human Services, Bethesda, Maryland, United States of America; 5 Laboratory of Hydrogeology and Hydroecology, Institute for Nature Management of the National Academy of Sciences, Minsk, Belarus; 6 Department of Oncology, Belarusian Medical Academy for Postgraduate Education, Minsk, Belarus; 7 Project Chernobyl, Brooklyn, New York, United States of America; 8 Department of Global Health, Medicine and Welfare, Atomic Bomb Disease Institute, Nagasaki University, Sakamoto, Nagasaki, Japan; 9 Department of Radiation Medical Sciences, Atomic Bomb Disease Institute, Nagasaki University, Sakamoto, Nagasaki, Japan; 10 Clinic and Polyclinic of Nuclear Medicine, University of Wuerzburg, Wuerzburg, Germany; University Hospital Basel, SWITZERLAND

## Abstract

One of the major health consequences of the Chernobyl Nuclear Power Plant accident in 1986 was a dramatic increase in incidence of thyroid cancer among those who were aged less than 18 years at the time of the accident. This increase has been directly linked in several analytic epidemiological studies to iodine-131 (^131^I) thyroid doses received from the accident. However, there remains limited understanding of factors that modify the ^131^I-related risk. Focusing on post-Chernobyl pediatric thyroid cancer in Belarus, we reviewed evidence of the effects of radiation, thyroid screening, and iodine deficiency on regional differences in incidence rates of thyroid cancer. We also reviewed current evidence on content of nitrate in groundwater and thyroid cancer risk drawing attention to high levels of nitrates in open well water in several contaminated regions of Belarus, i.e. Gomel and Brest, related to the usage of nitrogen fertilizers. In this hypothesis generating study, based on ecological data and biological plausibility, we suggest that nitrate pollution may modify the radiation-related risk of thyroid cancer contributing to regional differences in rates of pediatric thyroid cancer in Belarus. Analytic epidemiological studies designed to evaluate joint effect of nitrate content in groundwater and radiation present a promising avenue of research and may provide useful insights into etiology of thyroid cancer.

## Introduction

The most significant health consequence of the 1986 Chernobyl nuclear power plant accident was a dramatic increase in incidence of thyroid cancer among residents of Belarus, Ukraine, and parts of the Russian Federation affected by radioactive fallout. There is now compelling evidence for a causative association between excess of thyroid cancer and exposure to iodine-131 (^131^I) from the accident among those exposed in childhood or adolescence [1–9]. Along with young age at exposure, epidemiological studies have also suggested a modifying role of iodine deficiency on ^131^I-related risk of thyroid cancer [2, 10]. In this article, we discuss evidence for several confounding and modifying factors which may have affected the incidence of pediatric thyroid cancer in different regions of Belarus following the Chernobyl accident and propose a hypothesis that exposure to nitrates in drinking water due to usage of nitrogen fertilizers might influence the ^131^I-related risk.

## Materials and Methods

We performed searches in PubMed to identify studies of radiation-related risk of thyroid cancer, effect of thyroid screening, iodine deficiency and nitrate exposure on incidence of thyroid cancer overall and among populations exposed to the Chernobyl fallout. We reviewed reports prepared by major international bodies such as International Atomic Energy Agency (IAEA), United Nations Scientific Committee on the Effects of Atomic Radiation (UNSCEAR), and World Health Organization (WHO). We also relied on reviews of Belarusian journals and historical review publications in Russian language, as well as materials in German. Given the limited number of studies of iodine deficiency, thyroid screening, and nitrate exposure and little overlap among these and studies of radiation-related thyroid cancer, we summarized the state of scientific evidence and existing research needs. Studies were categorized as those providing estimates of radiation risk in the context of the Chernobyl accident, thyroid screening programs in Belarus, iodine intake and thyroid health and nitrates exposure and thyroid health.

Maps of contamination of Belarus with radioiodine and nitrate were generated with the ArcGIS Spatial Analyst extension of ArcGIS 10.2 software (Esri, Redlands, CA, USA) using previously published data [11] or the database of Laboratory of Hydrogeology and Hydroecology (Institute for Nature Management of the National Academy of Sciences of Belarus, Minsk, Belarus), respectively.

Statistical analyses were conducted using IBM SPSS Statistics Version 21 software package (International Business Machines Corp., Armonk, NY, USA). The rates of thyroid cancer were described using the negative binomial regression models with the logarithm as the link function as implemented in GENLIN procedure. *P*-values were 2-sided and considered significant if < 0.05.

## Results

### Thyroid cancer and radiation exposure

External beam therapy received in childhood has been known for decades to be associated with an elevated risk of developing thyroid cancer. The data come from a number of sources, but particularly from the radiotherapy for tinea capitis [12], enlarged thymus [13] or tonsils [14]. In addition, thyroid cancer was the first type of solid cancer to be found in excess among the survivors of the atomic bombings in Japan [15]. The best estimate of risk for persons exposed under 15 years of age to external radiation is based on a pooled analysis of seven studies [16]. The overall excess relative risk (ERR) among those exposed under 15 years of age was 7.7 per Gy, while the excess absolute risk (EAR) was 4.4 per 10^4^ person-years per Gy (PY Gy); linearity best described the dose response down to 100 mGy [16]. The ERR per Gy (ERR/Gy) significantly varied by age at exposure even within this limited age range (<15 years), with children exposed under one year of age having ERR/Gy five times higher than children exposed at 10–14 years. The excess risk peaked 15–19 years after radiation exposure, then declined, although an excess was still apparent more than 40 years later. Similar age at exposure pattern of ERR was observed in updated pooled analysis of thyroid cancer incidence following radiotherapy for childhood cancer [17]. In a recent analysis of thyroid cancer incidence from 1958 to 2005 among the atomic bomb survivors from Japan the ERR/Gy, modelled for those exposed at age 10 and observed at age 60, was 1.28 and the EAR was 3.0 [18]. It was estimated that the ERR decreased by 53%, while the EAR decreased by 70% per decade increase in age at exposure. Thyroid exposure to lower doses of external radiation (average of 120–260 mGy) from treatment for skin hemangioma in infancy was associated with significant increase in risk of thyroid cancer in two Swedish [19–20] and one French cohort [21] followed up to 39 years.

Ecological studies of thyroid cancer incidence in Belarus and Ukraine after the Chernobyl accident estimated a linear ERR/Gy of 18.9 and EAR/10^4^ PY Gy of 2.66 [6]. Case-control studies with individual dose estimates of ^131^I reported ERR/Gy between 4.2 and 7.2 among exposed children from Belarus and Russia [2–3, 22]. Cohort studies with measurement-based individual thyroid dose estimates reported ERR/Gy of 5.25 and 2.15 for prevalent thyroid cancer in Ukraine [8, 23] and Belarus [9, 24], respectively; and of 1.91 for incident thyroid cancer in Ukraine [1]. Another cohort study conducted in the Russian Federation estimated ERR/Gy of 3.22 [4]. The ERR/Gy estimates in these studies appear to be generally comparable with each other as well as with the estimate of radiation risk from the pooled study of external exposures [16]. In addition, similar to the pooled analysis, some but not all post-Chernobyl studies found increasing ^131^I thyroid cancer risk with decreasing age at exposure, which once again attests to the importance of the relationship between radiation risk and age at exposure [6–7, 25].

In Belarus, the rates of pediatric thyroid cancer began increasing as soon as four years after the Chernobyl accident [26]. The increase in incidence (Table 1) seemed to be region specific and generally depended on estimate of mean thyroid dose of ^131^I [27]. The highest thyroid dose (320 mGy) and incidence of pediatric thyroid cancer (11 per 100,000 PY) were in Gomel Oblast (an oblast is the largest administrative unit in Belarus, there are 6 oblasts in the country), while the lowest thyroid dose (3.5 mGy) and incidence were in Vitebsk Oblast (0.48 per 100,000 PY). There was one notable exception to the general dose-incidence rate pattern, i.e. substantial difference in rates of pediatric thyroid cancer in Mogilev and Brest Oblasts (1.50 and 5.51 per 100,000 PY, respectively) given generally comparable estimates of ^131^I doses in the two regions (65 and 51 mGy, respectively). This requires better understanding.

**Table 1:** Mean thyroid dose from ^131^I, nitrate in groundwater, number of pediatric thyroid cancers, and incidence rate of pediatric thyroid cancer among Belarusians 0–18 years old at the time of the Chernobyl accident.

Oblast	Mean ^131^I thyroid dose^a^ (mGy)	Nitrate in groundwater (mg/L)	Number of pediatric thyroid cancers	Incidence rate^b^ (per 100,000 PY)
Minsk^c^	26	130	114	1.20
Gomel	320	112	552	11.0
Mogilev	65	40	56	1.50
Brest	51	185	245	5.51
Grodno	11	53	58	1.74
Vitebsk	3.5	77	19	0.48
Belarus	78	-	1,044	3.48

^a^ Adapted from [27].

^b^ Crude incidence rates for 1986–2004 period.

^c^ Including the city of Minsk.

### Thyroid cancer and ultrasound screening

In addition to radiation dose, the contribution of ultrasound screening for early detection of thyroid cancer requires consideration as it could confound comparison of rates between regions with different levels of radiation exposure. Previous post-Chernobyl studies suggest that, due to enhanced surveillance or screening, thyroid cancer incidence rates may increase by a factor of 3–7 [4, 7, 25, 28].

The results of screening studies conducted in Belarus after the Chernobyl accident are summarized in Table 2. The first screening in the Gomel Oblast by the International Chernobyl Project (ICP) began in early 1990s under the supervision of the International Atomic Energy Agency [29]. The overall prevalence of nodular goiter in the study was 1.2%; the prevalence did not vary between children exposed and not exposed to radiation and no cases of thyroid cancer among 323 exposed children were found [30].

**Table 2:** Results of ultrasound screening programs for thyroid nodules and thyroid cancer in Belarus after the Chernobyl accident.

				Diseases			
Screening programs [reference]	Region	Years of screening	N	Thyroid nodules		Thyroid cancer	
				N	%	N	%
International Chernobyl Project, IAEA [30]	NA	1990	323	4	1.2	-	-
Screening program of Research Institute of Radiation Medicine [31–32]	Khoyniki (Gomel)	1990–1991 1993	1,132 1,546	14 32	1.2 2.1	7 5	0.6 0.3
International Program on Health Effects of the Chernobyl Accident (IPHECA), WHO [33]	Gomel Vitebsk Minsk	1990–1994	6,946 1,429 214	70 3 1	1.0 0.2 0.4	15 - -	0.2 - -
Chernobyl Sasakawa Health and Medical Cooperation Project [34]	Mogilev Gomel	1990–2000	13,868 19,790	24 350	0.1 1.7	2 38	0.008 0.2
Study of thyroid cancer and other thyroid diseases after the Chernobyl accident, NCI-Belarusian Ministry of Health, Gomel Oblast, Mogilev Oblast, Minsk city [9]	Gomel Mogilev Minsk-city	1996–2001	11,970	-	-	85	0.4
Screening project of the Red Cross^a^	Brest	1998–2005	50,896	3,244	6.4	136	0.3

^a^ Sivuda V., Grigorovich A., personal communication, Minsk, Belarus, 2013.

In 1990–1991, a thyroid screening of children in several contaminated areas of Gomel Oblast, conducted by the Research Institute of Radiation Medicine (Minsk, Belarus) found the overall prevalence of thyroid nodular disease in Khoiniki raion, one of the most contaminated raions, of 1.2% and of thyroid carcinoma—0.6%. The repeated screening of this population in 1993 showed that the proportion of thyroid nodules was 2.1% and the proportion of thyroid carcinoma was 0.3% [31, 32, 35].

In 1990, the WHO International Program on Health Effects of the Chernobyl Accident (IPHECA) examined about 8,000 children from Gomel, Mogilev, Vitebsk and Minsk Oblasts. The prevalence of thyroid nodules and thyroid cancer in Gomel Oblast was 1.0% and 0.2% respectively. In Vitebsk Oblast, the prevalence of nodular goiter was 0.2%, while no cases of thyroid cancer were found. In Minsk Oblast, similarly, the prevalence of nodular goiter was 0.4% and no single case of thyroid cancer was found [33].

Since 1990, a thyroid screening by the Chernobyl Sasakawa Health and Medical Cooperation Foundation (SMHF) Project of more than 19,000 children <18 years old at the time of the Chernobyl accident from Gomel Oblast and about 14,000 children from Mogilev Oblast reported the prevalence of nodular goiter in Gomel of 1.7% and that of thyroid cancer of 0.2%. The respective numbers in Mogilev Oblast were substantially lower, 0.1% and 0.008%, respectively [34].

In 1996, a cohort study of thyroid cancer and other thyroid diseases in Belarus after the Chernobyl accident was initiated by the Division of Cancer Epidemiology and Genetics, National Cancer Institute (USA) [36]. Overall, about 12,000 cohort members aged 18 years or younger at the time of the Chernobyl accident from the most contaminated regions of Gomel, Mogilev or from Minsk city were screened. The overall prevalence of thyroid cancer in this cohort was 0.4% [9].

The Red Cross is conducting a thyroid screening in Brest Oblast. Between 1998 and 2005, a total of 50,896 subjects aged less than 18 years at the time of the accident were examined. The overall prevalence of nodular goiter was 6.4% and of that of thyroid carcinoma—0.3%.

In summary, the comparison of data obtained by different investigators in 1990–2013 showed variations of thyroid cancer prevalence between 0.2%-0.6% in Gomel, 0.3% in Brest, and only 0.008% in Mogilev Oblast. Thus, the results of ultrasound screening programs suggest that given comparable screening, the difference in the prevalence of thyroid cancer between Brest and Mogilev Oblasts persists.

### Thyroid and iodine deficiency

Iodine is a critical element for thyroid hormone biosynthesis. The daily intake of iodine recommended by WHO and other international organizations ranges from 90 μg for young children to 150 μg for adolescents and adults [37]. Insufficient intake of dietary iodine is known to cause goiter, hypothyroidism, and mental retardation in children [38]. While animal experiments found that iodine deficiency may play a role in tumor promotion [39], the role of iodine deficiency in etiology of spontaneous thyroid cancer in humans has not been established unequivocally [40]. Normally, water and food are the main sources of iodine. Their iodine content depends on the iodine concentration in local soil and could vary within geographic regions. In rural areas, consumption of locally produced food is naturally higher than in urban populations. Also, consumption of water from open well sources is rather common. Therefore, rural residents are likely to be more vulnerable to iodine deficiency.

The territories affected by the Chernobyl accident had historically been an area of mild to moderate iodine deficiency [41]. Although there were several attempts to correct this situation in the decades preceding the accident, in the late 1980s before the breakup of the former Soviet Union, there were no systematic, large-scale iodination programs in place [41–42]. There are two ways in which iodine nutrition might affect the tumorigenic or oncogenic risk from exposure to radioactive iodine: by influencing the radiation dose received by the gland and by altering the response to the irradiation. This is due to the fact that iodine deficiency increases iodine uptake by the thyroid (including radioiodines, if present in the environment) and stimulates thyroid cell activity through increased production of thyroid stimulating hormone [42]. Consistent with this idea, two epidemiological studies, one conducted in Belarus and another one in the Russian Federation, found that the risk of radiation-related thyroid cancer was two-three times higher in iodine-deficient areas than in areas with normal iodine intake [2, 10]. In general, it is challenging to study the role of iodine deficiency in radiation-related thyroid cancer because data on iodine intake around the time of exposure, the most relevant time period, is often unavailable.

**Fig 1:**
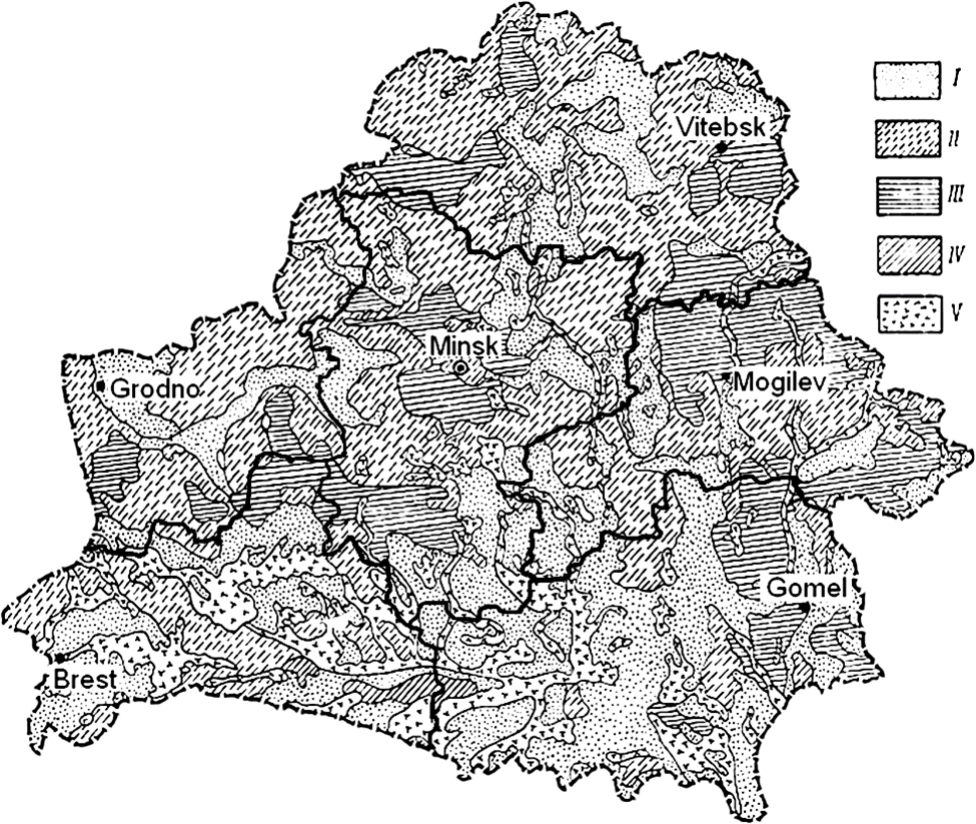
Content of iodine in soil of Belarus (mg/kg). I: 0.56–0.64; II: 0.87–0.94; III: 1.3–1.39; IV: 1.6–4.2; V: 5.0–18.2. The original map is derived from ref. [43] and is presented with minor modifications.

For evaluation of iodine status in Belarus before and immediately after the Chernobyl accident, the only source of data is measurements of iodine content in soil made in the 1960s [43]. Given that a substantial proportion of diet in Belarus in 1986 came from locally produced food and that governmental iodination programs in the 1980s were no longer active, the content of iodine in soil may provide useful insight on geographical differences in rates of pediatric thyroid cancer. According to level of iodine content, the territory of Belarus can be sub-divided into the following categories (Fig 1): 0.56–0.64, 0.87–0.94, 1.3–1.39, 1.6–4.2, and 5.0–18.2 mg/kg. Although high variation in soil iodine was observed in Gomel and Mogilev Oblasts, the majority of territory in these oblasts was characterized by low iodine content ranging between 0.56 mg/kg and 1.39 mg/kg. Soil iodine distribution in Brest Oblast was very uneven and patchy: areas with relatively high content of iodine (5.0–18.2 mg/kg) coexisted with low content areas (0.56–0.64 mg/kg), however higher iodine content in soil was observed generally in larger areas of Brest Oblast than in Gomel or Mogilev Oblasts (Fig 1).

Several population-based studies of urinary iodine concentration were conducted in Belarus in the 1990s. Iodine nutrition in a pediatric population is considered adequate if median iodine concentration is more than 100 μg/L, mildly iodine deficient if it is 50 to 99 μg/L, moderately deficient if 20 to 49 μg/L, and severely deficiency if less than 20 μg/L [44]. Five years after the accident, the SMHF project measured urinary iodine concentration in 5,710 children [45] from Chernobyl contaminated territories and reported that median iodine concentrations in children from Gomel and Mogilev Oblasts were high and comparable to each other (169 and 177 μg/L, respectively). Another study, a 1995–1998 survey of 11,562 children, reported median urinary iodine concentrations of 79.8, 49.0 and 27.3 μg/L in Gomel, Mogilev and Brest Oblasts, respectively [46]. Measurements of urinary iodine concentration in the Belarusian-American cohort conducted in 1997–2001 are consistent with those reported by Arinchin et al. The median iodine concentration in Gomel (69 μg/L) was higher than in Mogilev (39 μg/L) [47]. No children from Brest Oblast were examined either by the SMHF or Belarusian-American study. In interpretation of urinary iodine data, however, one should keep in mind that measurements of urinary iodine concentrations performed in the 1990s may not necessarily reflect the situation with iodine intake at the time of the Chernobyl accident, as some stable iodine supplementation programs were implemented in contaminated areas of Belarus, especially in Gomel Oblast.

In summary, using soil content of iodine as a surrogate indicator of regional iodine status around the time of the Chernobyl accident, Brest Oblast does not appear to be more iodine deficient than either Gomel or Mogilev Oblast. This coupled with slightly lower mean thyroid doses from ^131^I cannot explain higher incidence rates of pediatric thyroid cancer in Brest compared to Mogilev Oblast (Table 1), although the role of individual iodine intake cannot be ruled out.

### Thyroid diseases and nitrate pollution

Nitrate is a common contaminant of drinking water, particularly in agricultural areas, due to the usage of nitrogen-containing fertilizers introduced in the 1950s, contamination from refuse dumps, oxidation of ammonia from human and animal waste, and treatment of drinking water with chloramines. High amounts of nitrate might also be present in some fruits and vegetables as a result of cultivation in greenhouses, and in cured and processed meats due to their addition as preservatives or color enhancers. Medications, including antidiarrheals, diuretics, and vasodilators, also contribute to nitrate exposure in humans [48].

Nitrate is known to affect the metabolism of iodine in the thyroid. Ingested nitrate may act as an endocrine disruptor by binding to the sodium-iodide symporter on the apical surface of thyroid follicular cells and thus interfering with thyroidal uptake of iodide. As a result, the levels of triiodothyronine (T3) and thyroxin (T4) decrease, while the levels of thyroid stimulating hormone (TSH), through a negative feedback loop, increases. Chronic stimulation of the thyroid by TSH can lead to proliferative changes, including hypertrophy and hyperplasia as well as neoplasia [49–50]. Consistent with this idea, an experimental study by Eskiocak et al. showed that, following a 30-week period of oversupply of nitrate to rats, uptake of iodine by rats’ thyroid increased in the 250 mg/L and 500 mg/L nitrate groups while it was decreased in the 50 mg/L group as compared to controls [51]. The corresponding thyroid gland weight increased in all nitrate-fed animal groups. These results suggest that chronic exposure to high levels of nitrate, similarly to the effect of iodine deficiency, might result in the development of goiter through TSH stimulation. There are other mechanisms by which ingested nitrate may produce detrimental effects on health [52]. One is through formation of methemoglobin that inhibits the oxygen-carrying capacity of blood, and another is through endogenous formation of *N*-nitroso compounds that may act as the carcinogens [53].

Drinking water is likely to be a primary source of nitrate exposure when nitrate concentrations are > 50 mg/L [53]. Several studies from Slovakia, Bulgaria, Germany and the USA reported associations between nitrate exposure and thyroid function as well as thyroid cancer in humans [54–59]. The risk of goiter in Bulgarian schoolchildren [54] and pregnant women [55] exposed to a high level of nitrate in drinking water (75 mg/L) was significantly higher than in schoolchildren and pregnant women exposed to low levels of nitrate (8 mg/L). In addition, the risk of thyroid disorders in pregnant women living in the village with high nitrate levels in drinking water was five times higher than that of ‘non-exposed’ women [55]. Studies of Slovak schoolchildren [57–58] similarly found that thyroid volume estimated by ultrasound was significantly higher in children from high nitrate area (51–274 mg/L) than in children of comparable age from low nitrate area (< 2 mg/L). While no differences in the levels of T4 or T3 were found, the frequency of TSH level in the range of subclinical hypothyroidism (> 4.0 mIU/L) and that of positive antibodies to thyroid peroxidase was higher in children from high nitrate area [57]. Based on a random sample of adults between 18 and 70 years old from all over Germany, Hampel at al. found a weak, but significant correlation between nitrate content in urine and thyroid size among persons with a nitraturia of > 60 mg NO_3_^-^/g of creatinine [56]. Together, the results of these studies confirm the role of high-nitrate level in drinking water as a risk factor for thyroid dysfunction in vulnerable population groups. Long-term exposure to nitrate in drinking water has been also evaluated in relation to multiple cancer sites and positive associations were reported for cancers of the esophagus, stomach, bladder, and colon [53]. With regard to thyroid cancer, Ward et al. found an increased risk for higher average nitrate levels in public water supplies and for longer consumption of water exceeding 5 mg/L nitrate (for ≥5 years at >5 mg/L), OR = 2.6 (95% CI, 1.1–6.2) [59]. The authors observed no association with the prevalence of hypothyroidism or hyperthyroidism. Higher intake of dietary nitrate was also associated with an increased risk of thyroid cancer (*P*-value for trend = 0.046) and with the prevalence of hypothyroidism (*P*-value for trend = 0.001), but not of hyperthyroidism [59].

In Belarus, between 1960 and 1990, the use of nitrogen fertilizers increased 20–25 times from 4 to 92 kg/hectare, while the average level of nitrate in ground water increased from 1.1 to 41.6 mg/L [60]. According to the Belarusian Ministry of Health, the proportion of pipeline water samples with nitrate concentration exceeding the WHO-recommended Maximum Contaminant Level (MCL) of 45 mg/L is about 1% [61]. In contrast, about 40% of water samples from open wells have nitrate concentration exceeding the MCL. In Brest and Gomel Oblasts, the proportion of such samples reaches 40–60%, while in Mogilev Oblast it is about 20% [61]. Concentration of nitrate in groundwater in the early 1990’s was 112 mg/L in Gomel, 40 mg/L in Mogilev, and 185 mg/L in Brest Oblast (Table 1) exceeding the MCL by 2.5 and 4.0 times in Gomel and Brest, respectively. Groundwater from open wells is the main source of drinking water in rural areas in Belarus.

**Fig 2:**
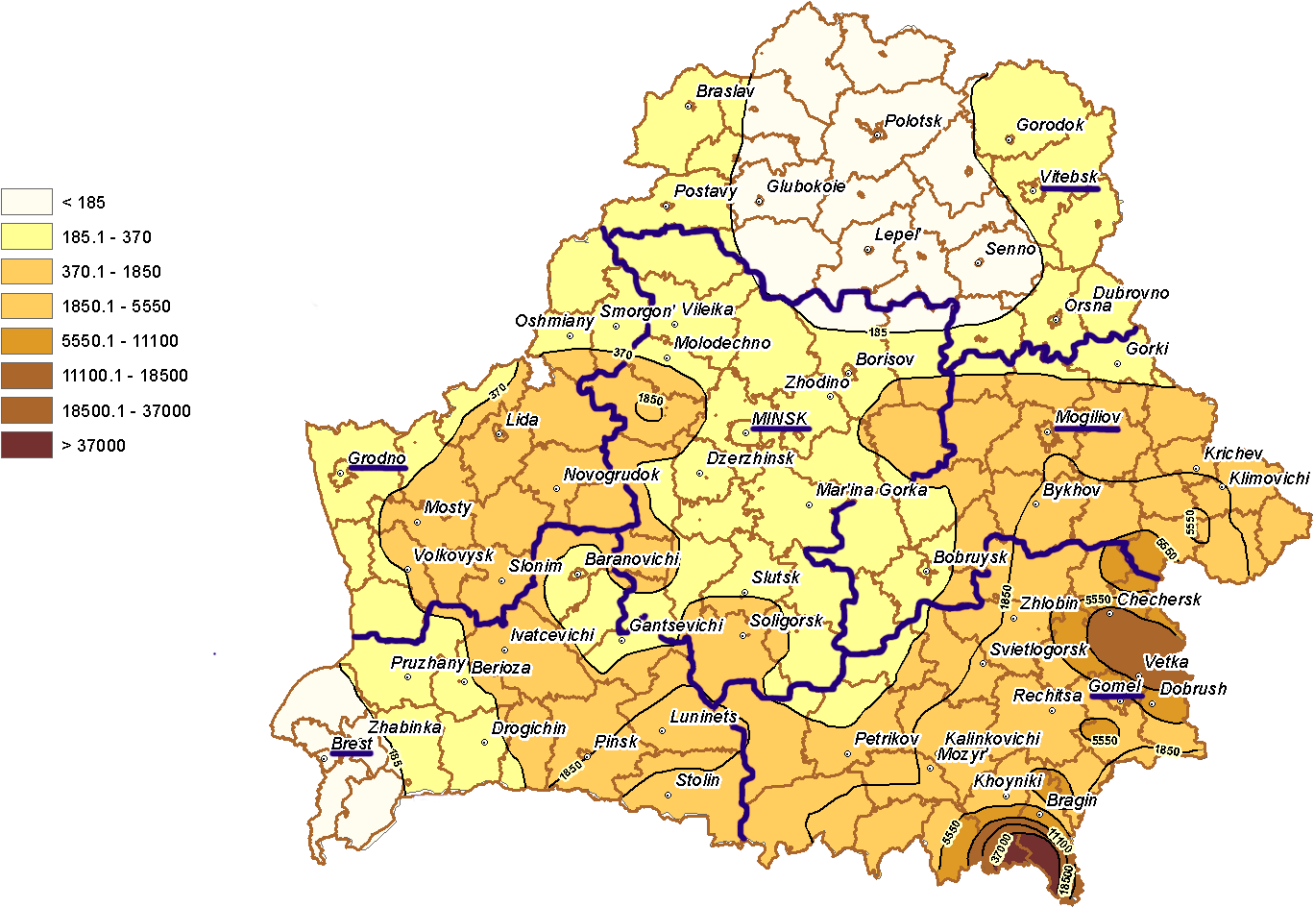
^131^I deposition density (kBq/m^2^) in the soil of Belarus as of May 10, 1986 [11]. Borders and administrative centers of oblasts are highlighted in violet.

**Fig 3:**
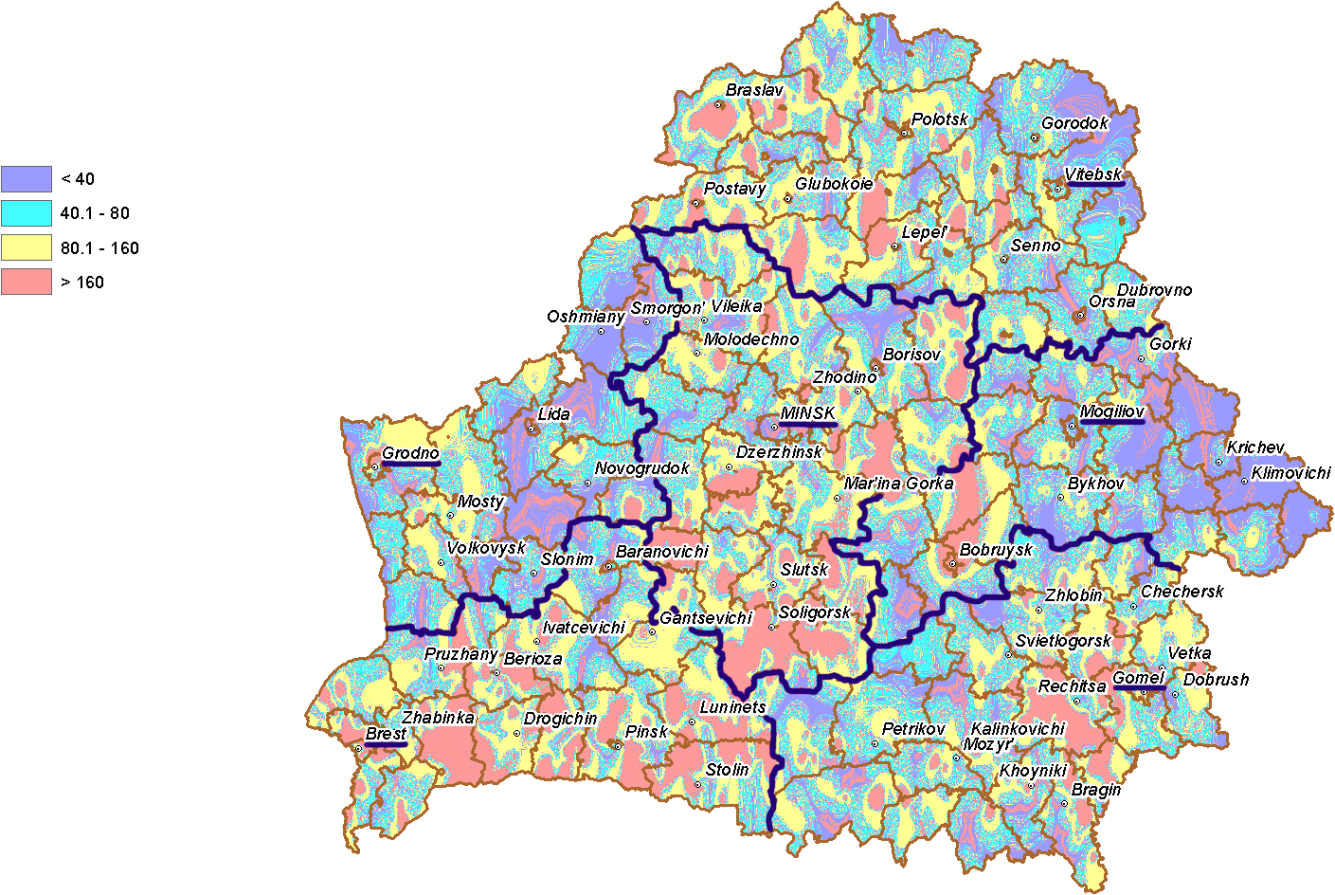
Nitrate concentration (mg/L) in groundwater from open wells in different areas of Belarus in 1988–1990. Borders and oblast administrative centers are highlighted in violet.

The comparison of two maps, i.e. ^131^I deposition density (Fig 2) and nitrate concentration in groundwater well water (Fig 3), with estimates of incidence of pediatric thyroid cancer (Table 1) suggests that the differences in incidence rates among Gomel, Brest and Mogilev Oblasts may be related not only to exposure to ^131^I but also to the nitrate levels in groundwater.

**Fig 4:**
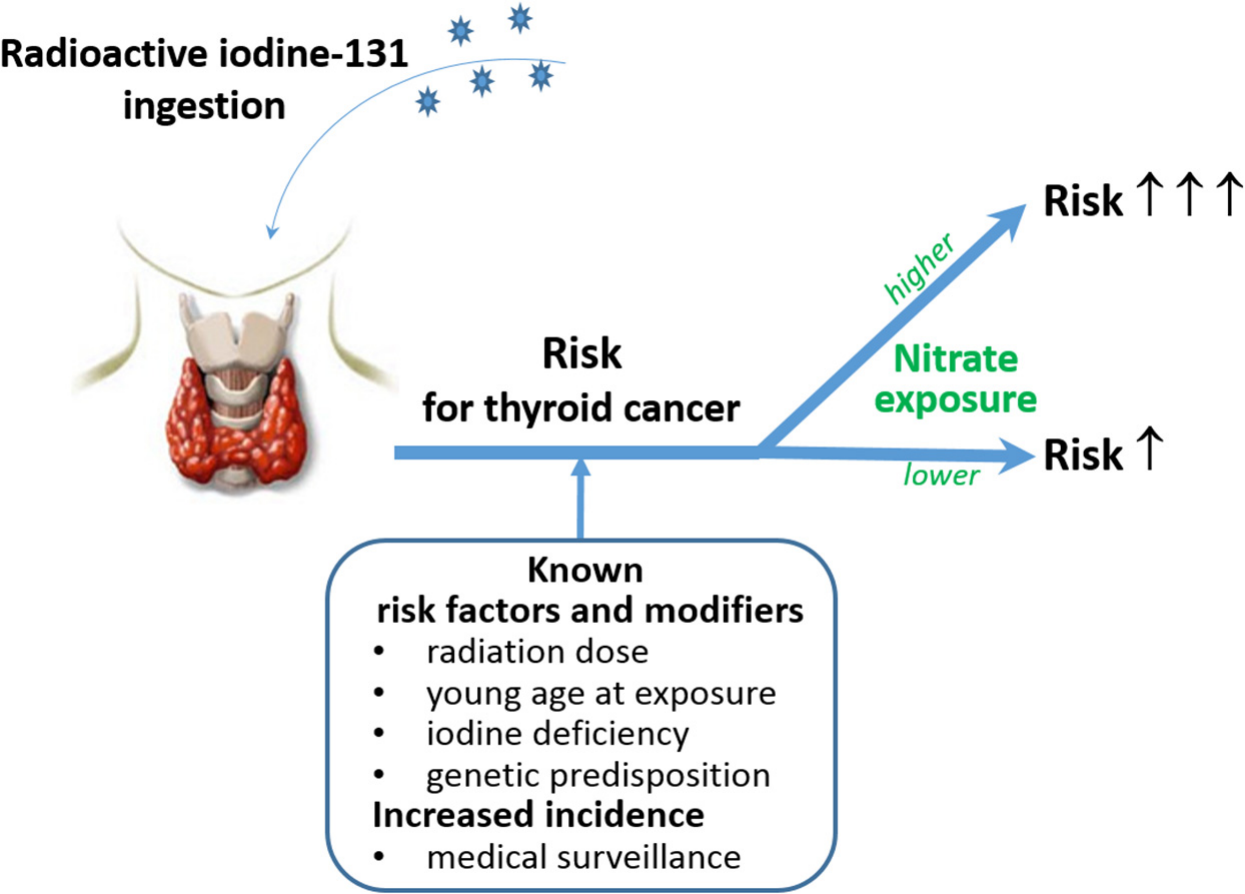
Recapitulation of the combination effect of radiation and nitrates on thyroid cancer risk. Exposure to both radiation and high levels of nitrates is proposed to increase risk for developing thyroid cancer.

We assessed the individual and joint effect of mean thyroid dose due to ^131^I and mean nitrate concentration in groundwater for all oblasts of Belarus using negative binomial regression models (Table 3). Our analyses indicated that, in univariate models, radiation dose was significantly associated with thyroid cancer incidence (*P* = 0.029), while nitrate concentration was not (*P* = 0.301). When radiation dose and nitrate concentration were included in the model simultaneously, neither the magnitude of the effects nor their statistical significance changed meaningfully compared to univariate models. This implies radiation dose and nitrate pollution do not counfound each other. At the same time, we found that the effect of radiation significantly varied according to nitrate concentration (*P* = 0.004). A plausible interpretation is that, at a regional level, radiation effect might be modified by or depend upon the level of nitrates in drinking water (recapitulated in Fig 4). This finding must be considered preliminary, however, because of intrinsic limitations of ecological studies and the usage of aggregate rather than individual data. Further analytic epidemiological studies are required to quantify the effect of nitrate pollution on risk of developing thyroid cancer after radiation exposure.

**Table 3:** Relationship between childhood thyroid cancer incidence, radiation thyroid dose and nitrate in groundwater in Belarus.

Predictor	Deviance	Degrees of freedom	*P*-value
None^a^	6.190	5	
Dose	2.539	4	0.029^b^
Nitrate	4.761	4	0.301^b^
Dose + Nitrate	1.355	3	0.002^b^
(Dose)			0.007^c^
(Nitrate)			0.125^c^
Nitrate + Dose*Nitrate^d^	1.321	3	0.001^b^
(Nitrate)			0.305^c^
(Dose*Nitrate)			0.004^c^

^a^ Intercept-only model.

^b^ Likelihood-ratio chi-square test of the current model fit comparing to an empty model.

^c^ Significance of the regression coefficient associated with a given predictor in the current model, Wald chi-square test.

^d^ A multiplicative interaction term between radiation dose and nitrate concentration.

## Conclusion and Research Needs

The worldwide incidence of thyroid cancer steadily increased during the last 30–40 years [62–63], mainly attributed to the growing incidence of papillary thyroid carcinoma (PTC). PTC is the most common histological type of thyroid cancer, for which rates in the USA changed from 4.3 per 100,000 PY in 1973–1974 to 11.4 per 100,000 PY in 2005–2006 [64]. The main factors contributing to this increase continue to be debated. One of these is related to the extensive use of ultrasound (first introduced in 1980s) for diagnosis of structural thyroid diseases. Consistent with this idea, incidence of small thyroid cancers best discovered with new technologies such as ultrasound and fine-needle aspiration biopsy, have risen particularly sharply. However, increased medical surveillance and more sensitive diagnostic procedures do not completely explain the increase in rates of PTC since a significant increase is also observed for larger tumors (>10 mm) [65]. Thus, further investigation is required to determine additional factors contributing to the growing incidence of PTC as well as whether these might be associated with specific types of somatic mutations (e.g., *BRAF* or *RAS* point mutations, chromosomal rearrangements, *CLIP2* amplification).

Our hypothesis generating work focused on evaluation of incidence rates of pediatric thyroid cancer in Belarus in relation to levels of radiation exposure, thyroid cancer screening, iodine deficiency and nitrates in groundwater. The Belarusian data at a regional level suggest that nitrate content in drinking water may affect the rates of thyroid cancer in irradiated populations. We realize that, similar to other ecological studies, which combine demographic and geographic information, the lack of individual data is a major limitation of this evaluation. Other endocrine disruptors and carcinogens such as heavy metals, pesticides, perchlorate, thiocyanate and solvents may also occur in drinking water as a consequence of human activities and natural hydrogeochemical processes, but could not be evaluated in our study. We focused on nitrate contamination of drinking water because for Belarus it is a more important problem, particularly in rural areas where majority of population exposed to radioiodines from the Chernobyl accident continues to reside.

In conclusion, due to the increasing rates of thyroid cancer and growing use of medical radiation for diagnostic and therapeutic purposes, coupled with biological plausibility of the effects of nitrates on the thyroid, there is a need for analytic epidemiological studies designed to evaluate a joint effect of nitrate exposure and radiation on risk of thyroid cancer at an individual level. Ideally, the epidemiology, pathology, genetic and molecular data should be combined. Such integrative studies may provide useful insights into the etiology and molecular mechanisms of thyroid cancer.
